# Patterns of medical comorbidities among aging people receiving heroin-assisted treatment: A 10-year single-center repeated cross-sectional study

**DOI:** 10.1016/j.dadr.2026.100435

**Published:** 2026-04-01

**Authors:** David Strittmatter, Dario Willi, Peer W. Brandt, Sonja Loebnitz, Eveline Hofmann, Maria Christine Thurnheer

**Affiliations:** a Department of Infectious Diseases, Inselspital, Bern University Hospital, University of Bern, Switzerland; b KODA Bern, Behandlungszentren für Suchtmedizin BZS, Belpstrasse, Bern 3007, Switzerland

**Keywords:** Non-communicable diseases, People who use drugs, Harm reduction, Integrated care, Access to health care, Opioid agonist treatment, Heroin agonist treatment

## Abstract

**Background:**

This study examined the patterns of medical comorbidities among aging people who use drugs (PWUD) enrolled in a heroin-assisted treatment (HAT) program in Bern, Switzerland, over ten years.

**Methods:**

Cross-sectional assessments were conducted at a single HAT center in 2009 (n = 200), 2012 (n = 215), and 2019 (n = 203). A group of long-term participants (n = 102) present at all three time points was identified and compared to other participants over time. Data on demographics; infectious, psychiatric, and medical comorbidities; substance use; and opioid agonist treatment (OAT) were analyzed using descriptive statistics and logistic regression.

**Results:**

In 2019, the median age of long-term participants was 52 years, one-third of patients were women. Hepatitis C virus (HCV) RNA positivity declined from 50/102 (49%) in 2009 to 10/102 (9.8%) in 2019, whereas HIV prevalence remained stable at 12% with high treatment uptake. Medical multimorbidity (≥2 medical comorbidities) increased from 8.8% to 37.3% (*p* < 0.001), with cardiovascular and musculoskeletal diseases being the most common comorbidities. In 2019, non-long-term participants were younger (median 42 years) with lower multimorbidity (16.8% vs. 37.3%, *p* = 0.008). Ongoing use of non-prescribed drugs (odds ratio (OR) 2.49; 95% confidence interval (CI) 1.17–5.28; *p* = 0.018) and advancing age (OR 1.08; 95% CI 1.03 – 1.14; *p* = 0.002) independently predicted multimorbidity in 2019.

**Conclusions:**

Over 10 years, prevalence of active HCV infection among PWUD in long-term HAT declined, whereas non-communicable diseases increased. Integrated care models combining OAT with medical services are essential to meet the evolving health needs of this population.

## Highlights

• Prevalence of active HCV infection declined over 10 years among PWUD in heroin-assisted treatment (HAT).
• Medical multimorbidity increased substantially in long-term HAT participants.
• Older age and ongoing non-prescribed drug use independently predicted multimorbidity.
• Findings support integrated opioid agonist, medical, and psychiatric care models.

## 1 Introduction

The long-term health trajectories of people who use drugs (PWUD) are becoming a critical concern as the population ages. Advances in harm-reduction strategies and treatment options for Hepatitis C Virus (HCV) and Human Immunodeficiency Virus (HIV) have significantly reduced overall mortality and improved outcomes for infectious diseases among PWUD (Degenhardt et al., 2017, Graf et al., 2020, Grebely et al., 2017, Grebely et al., 2015, Keiser et al., 2018, Kimmel et al., 2021). Nonetheless, non-communicable diseases (NCDs) have emerged as a new healthcare challenge. NCDs including cardiovascular diseases, cancers, chronic respiratory diseases and diabetes, are chronic conditions not transmitted between individuals and typically progress slowly over time (Beaglehole et al., 2011). Globally, NCDs account for approximately three-quarters of all deaths and their prevalence increases substantially with age (Naghavi et al., 2025). Lewer et al. demonstrated that the prevalence of NCDs among PWUD is four-fold higher than that of drug-related conditions, with cardiovascular, respiratory, and liver diseases being the predominant causes of morbidity and mortality (Lewer et al., 2019).

Multimorbidity, defined as the presence of two or more chronic conditions in the same individual, globally affects roughly one third of adults with prevalence exceeding 50% among persons ≥ 60 years of age (Barnett et al., 2012, Chowdhury et al., 2023). In Switzerland, prevalence estimates for multimorbidity are 15.7% (CI 12.3.–19.1%) for persons aged 50–59 years, increasing substantially in older age groups (Rizza et al., 2012).

Heroin assisted treatment (HAT) is a specialized form of opioid agonist therapy in which diacetylmorphine is prescribed and administered under medical supervision to individuals with severe opioid disorder who have not responded adequately to conventional treatments such as methadone or buprenorphine (Csete et al., 2016, McNair et al., 2023)

In Switzerland, harm reduction programs, including needle and syringe exchange programs (NSP), OAT, and HAT, have helped combat drug-related mortality and the spread of HIV among people who inject drugs since the severe public health challenge associated with open drug scenes and rising HIV infections of the late 1980s (Fischer et al., 2007, Meyer et al., 2025, Rusch et al., 2019, Uchtenhagen, 2010). These programs have contributed to a significant decrease in drug-related mortality, as well as in the transmission rates of HCV and HIV. However, reductions in drug-related mortality have contributed to an aging population of PWUD with evolving healthcare needs, particularly those related to NCDs and multimorbidity. These emerging concerns remain insufficiently addressed in the published literature, and are challenging in clinical settings (Heidari et al., 2023, Heidari et al., 2024).

The present study addressed this gap by providing a detailed analysis of the healthcare needs of aging PWUD on HAT, with particular attention to the shift from infectious diseases to NCDs. Using sequential cross-sectional assessments at a single HAT center in Bern, Switzerland, we examined changes in healthcare needs among individuals engaged in long-term treatment. To assess the impact of ongoing drug use, harm reduction strategies and aging on the prevalence of NCDs, we compared this group with participants receiving HAT for a shorter duration. This approach aims to inform the development of integrated care models that address both infectious diseases and NCDs, supporting comprehensive healthcare for aging PWUD (Samet et al., 2001).

## 2 Methods

### 2.1 Study center and setting

The study was conducted in a Swiss OAT center (KODA Bern, Switzerland), certified to prescribe medical heroin (diacetylmorphine) and other types of opioids. Eligible patients were aged 18 years or older, had a history of opioid use for at least 2 years, and had previously not responded to at least two treatment attempts using opioid-assisted or abstinence-oriented approaches. Participants had to comply with regular meetings with nursing staff, social workers, and psychiatric evaluations. Patients on heroin treatment attended the center twice a day, seven days a week, during specified opening hours, for on-site, supervised injection, or oral intake of the prescribed substitution medication.

Starting in 2009, in collaboration with a tertiary care university hospital (Department of Infectious Diseases, University Hospital Bern, Switzerland), on-site medical care was offered in addition to established psychiatric treatment. Specialists in infectious disease and internal medicine from the tertiary care hospital conducted on-site clinics three times a week, complementing the existing psychiatric services. Early efforts focused on the diagnosis and treatment of HCV and HIV as most pressing medical concerns, nonetheless, management of NCDs was integral part of this care model.

### 2.2 Data source and collection

To assess the medical needs (both infectious disease and NCD related) of participants, we conducted sequential, single-center, cross-sectional surveys in 2009, 2012, and 2019 including all patients registered at the time of assessment. Due to the cross-sectional design, no information on patients exiting the program (lost to follow-up or death) was collected.

Data sources included patient medical history, electronic pathology results, prescription charts, social worker notes, and nursing records. The following parameters were collected: sociodemographic data (sex, date of birth, duration of stay in the program, information on current housing situation, legal issues where available, information on social networks), substitution therapy (substances currently prescribed), non-prescribed drug use, problematic alcohol consumption (yes, if formally diagnosed (> 4 standard units/day in males; > 3 standard units/day in females) or regular monitoring of breath alcohol levels was performed), psychiatric comorbidities (ICD-10 codes from patients' charts, most prevalent diagnoses (excluding F10, substance dependence syndrome) were assessed: F3 affective disorders, F6 personality disorders, F2 schizophrenia), medical comorbidities (ICD-10 codes from patient chart, further classified by organ system), serology data (transcribed from electronic pathology results), and information on HCV and HIV treatment initiation/completion. Multimorbidity was defined as the presence of two or more medical comorbidities (counted at the organ system level, see above) per patient, psychiatric comorbidities were evaluated separately and not included in this multimorbidity assessment. Psychiatric comorbidity was defined as the presence of at least one of the following: affective disorders (F3), personality disorders (F6), or schizophrenia (F2).

All pre-specified data were recorded with encoded identifiers using a standardized case report form. Data quality and accuracy were monitored using MCT, DS, and DW, with queries and data amendments performed as necessary.

### 2.3 Study population and statistical analysis

There were 200 active patients in 2009, 215 in 2012, and 203 treated at KODA in 2019.

A group of long-term participants including 102 patients present in all three assessments was identified and compared to the non-long-term patients at each survey.

A group of patients only registered in 2019 (short-term participants, n = 72) was identified and compared to long-term participants to examine the robustness of patterns observed in the main analysis (Supplementary Material).

For descriptive measures, categorical variables were summarized as counts and percentages, and numerical variables were summarized as means and standard deviations for normally distributed variables and as medians and interquartile ranges otherwise. For statistical inference, group comparisons were based on the chi-square or Fishers exact test for categorical outcomes, Student’s *t*-test for normally distributed quantitative outcomes, and the unpaired two-sample Wilcoxon test otherwise. A two-sided significance level of α= 0.05 was used throughout the study.

To adjust for false discovery rates with multiple testing, the Benjamini-Hochberg procedure was applied, assuming a false discovery rate of 0.1, where appropriate.

Multimorbidity was modelled as a binary outcome. Candidate predictors included time in treatment (per year increase), age (per year increase), HCV seropositivity, psychiatric comorbidity and non-prescribed drug use. Results are reported as odds ratios (ORs) with 95% confidence intervals (CIs) and corresponding p-values.

Variable selection for the multivariable model was guided by clinical reasoning and prior literature rather than solely by statistical significance in univariable analyses. Given the sample size (n = 203), the number of variables entered into the multivariable model was deliberately constrained to reduce the risk of overfitting (Greenland et al., 2016, Mickey and Greenland, 1989, Peduzzi et al., 1996, Sauerbrei et al., 2020, van Smeden et al., 2019). Multicollinearity among all included predictors was formally assessed. Variance Inflation Factor (VIF) and tolerance statistics were computed by fitting an ordinary least squares (OLS) regression model with the identical set of predictors, following standard recommendations (Kim, 2019, Vatcheva et al., 2016). VIF values exceeding 10 and tolerance values below 0.10 were predefined as indicative of serious multicollinearity (Kim, 2019, O’brien, 2007). Results of the multicollinearity diagnostics are presented in Supplementary Material, sTable 4. Statistical analyses were performed using STATA MP 18.

### 2.4 Ethical compliance

The study proposal was reviewed by the Ethics Commission of the Canton of Bern, Switzerland and was assessed as not requiring formal approval due to its purely observational design and the absence of study-related interventions (BASEC Nr. Req-2018–00855). All patients treated at the HAT center signed a general consent form for use of encoded data recorded during routine care.

## 3 Results

### 3.1 Characteristics of all registered patients at cross sectional surveys over time

During three surveys, 200, 215 and 203 patients were registered in 2009, 2012 and 2019 ((Table 1). One third of patients were women and the median age increased from 39.5 years in 2009 to 49 years in 2019. While medical heroin was the most frequent opiate prescription in 2009 and 2012 (163/200 (81.5%) and 199/215 (92.6%)), morphine was the leading OAT prescription in 2019 (187/203 (92.5%) on morphine and 150/203 (74.3%) on heroin), reflecting a shift in OAT strategies due to the availability of oral morphine preparations over time.

**Table 1:** Population characteristics over time, all participants.

Year of survey		2009	2012	2019	
		N = 200 (%)	N = 215 (%)	N = 203 (%)	*p*-value
*Demographics*					
Age, years		39.5 (36.0–43.0)	43.0 (38.0–47.0)	49.0 (41.0–54.0)	***0.002***
Time in KODA, years		6.1 (5.0–10.0)	8.4 (4.3–12.8)	13.7 (6.4–19.4)	***0.002***
Women		57 (28.5%)	64 (29.8%)	62 (30.5%)	*0.900*
*Psychiatric diagnosis*					
F3 Affective Disorders		40 (20.0%)	65 (30.2%)	67 (33.0%)	***0.015***
F6 Personality Disorder		38 (19.0%)	43 (20.0%)	71 (35.0%)	***0.002***
F2 Schizophrenia		9 (4.5%)	14 (6.5%)	36 (17.7%)	***0.002***
At least 1 psychiatric comorbidity (F3, F6 or F2)		76 (38.0%)	101 (47.0%)	111 (54.7%)	***0.007***
*Opiate agonist treatment*					
Heroin		163 (81.5%)	199 (92.6%)	150 (73.9%)	***0.002***
Methadone		81 (40.5%)	108 (50.2%)	33 (16.3%)	***0.002***
Morphine		0 (0.0%)	15 (7.0%)	187 (92.1%)	***0.002***
*Psychiatric co-medication*					
Benzodiazepines		38 (19.0%)	58 (27.0%)	75 (36.9%)	***0.002***
Antipsychotics		27 (14%)	46 (21%)	46 (23%)	*0.064*
Methylphenidate or Dexmethylphenidate		NA	9 (4.2%)	14 (6.9%)	*0.309*
Antidepressants		NA	58 (27.0%)	70 (34.5%)	*0.141*
*Additional substance use*					
Non-prescribed drug use		59 (29.5%)	81 (37.7%)	121 (59.6%)	***0.002***
Problematic alcohol use		41 (20.5%)	53 (24.7%)	55 (27.1%)	*0.380*
*Hepatitis C*					
HCV antibody positive		159 (79.5%)	163 (75.8%)	152 (74.9%)	*0.622*
	HCV RNA positive	104/159 (65.4%)	92/163 (56.4%)	29/152 (19.1%)	***0.002***
	HCV treated with SVR	8/159 (5.0%)	17/163 (10.4%)	76/152 (50.0%)	***0.002***
	HCV with spontaneous clearance	47/159 (29.6%)	54/163 (33.1%)	47/152 (30.9%)	*0.862*
*HIV*					
Diagnosed		24 (12.0%)	23 (10.7%)	19 (9.36%)	0.801
Treated if diagnosed		NA	22 (95.7%)	18 (94.7%)	0.872

All values are median (interquartile range) or number (%).

Abbreviations: HCV, hepatitis C virus; HIV, human immunodeficiency virus; SVR, sustained virological response. *p*-values in bold were significant after Benjamini Hochberg correction assuming a false discovery rate of 0.1.

Prevalences of diagnosed affective disorder (F3), personality disorder (F6) and schizophrenia (F2) increased over time (Affective disorder: 40/200 (20%) in 2009, 67/203 (33%) in 2019, *p* = 0.015; Personality disorders: 38/200 (19.0%) in 2009 and 71/203 (35%) in 2019, *p* = 0.002; Schizophrenia: 9/200 (4.5%) in 2009 and 36/203 (17.7%) in 2019, *p* = 0.002). Similarly, the proportion of patients with at least one psychiatric comorbidity (F3, F6 or F2) increased over time (76/200 (38.0%) in 2009; 111/203 (54.7%) in 2019, *p* = 0.007). Co-medication with benzodiazepines significantly increased over time (38/200 (19.0%) in 2009–75/203 (37.1%) in 2019, *p* = 0.002) while other psychiatric prescriptions remained relatively stable.

A marked increase in self-reported non-prescribed drug use was observed over time (59/200 (29.5%) in 2009 to 121/203 (59.6%) in 2019 *p* = 0.002), while problematic alcohol consumption remained relatively stable at around 25%. The prevalence of patients with antibodies against HCV remained stable at around 75% (159/200 (79.5%) in 2009, 163/215 (75.8%) in 2012 and 152/203 (74.9%) in 2019), while the proportion of HCV RNA positive individuals declined significantly (104/159 (65.4%) in 2009, 92/163 (56.4%) in 2012 and 29/152 (19.1%) in 2019, *p* = 0.002). Percentage of patients with sustained virologic response after treatment increased from 8/159 (5.0%) in 2009 to 76/152 (50%) in 2019, *p* = 0.002). The proportion of patients with spontaneous HCV clearance remained stable at around 30%.

The proportion of people living with HIV (PLHIV) remained stable (24/200 (12.0%) in 2009, 23/215 (10.7%) in 2012, 19/203 (9.36%) in 2019) with a high treatment rate of around 95%.

### 3.2 Characteristics of long-term-participants over time

We identified 102 patients (50.8% of all 200 patients treated in 2009) as long-term participants with documented information at all three assessments (Table 2 and Supplementary Material, sTable 1). In 2019, 29/102 long-term patients (28.4%) were women (one patient transitioned from female to male between 2012 and 2019). The median age of the long-term participants was 52 years in 2019, significantly higher than the 42 years of non-long-term participants (*p* = 0.007).

**Table 2:** Population characteristics over time, long-term participants compared to others.

Year of survey		2009			2012			2019		
Participant group		Long term	others		Long term	others		Long term	others	
		N = 102 (%)	N = 98 (%)	*p*-value	N = 102 (%)	N = 113 (%)	*p*-value	N = 102 (%)	N = 101 (%)	*p*-value
*Demographics*										
Age, years		40.0 (36.0–44.0)	39.0 (36.0–43.0)	*0.621*	44.0 (40.0–48.0)	42.0 (34.0–46.0)	***0.022***	52.0 (49.0–57.0)	42.0 (37.0–50.0)	***0.007***
Time in KODA, years		8.0 (5.0–11.2)	5.0 (5.0–8.0)	***0.014***	10.9 (7.7–14.0)	5.8 (2.8–10.7)	***0.011***	19.0 (16.0–22.2)	6.4 (3.2–10.8)	***0.007***
Women^a)^		30 (29.4%)	27 (27.6%)	*0.925*	30 (29.4%)	34 (30.1%)	*0.914*	29 (28.4%)	33 (32.7%)	*0.593*
*Psychiatric diagnosis*										
F3 Affective Disorders		25 (24.5%)	15 (15.3%)	*0.374*	27 (26.5%)	38 (33.6%)	*0.430*	35 (34.3%)	32 (31.7%)	*0.759*
F6 Personality Disorder		22 (21.6%)	16 (16.3%)	*0.621*	22 (21.6%)	21 (18.6%)	*0.644*	32 (31.4%)	39 (38.6%)	*0.421*
F2 Schizophrenia		3 (2.9%)	6 (6.1%)	*0.621*	4 (3.9%)	10 (8.8%)	*0.264*	15 (14.7%)	21 (20.8%)	*0.421*
At least 1 psychiatric comorbidity (F3, F6 or F2)		42 (41.2%)	34 (34.7%)	*0.621*	47 (46.1%)	54 (47.8%)	*0.840*	52 (51.0%)	59 (58.42%)	*0.421*
*Opiate agonist treatment*										
Heroin		91 (89.2%)	72 (73.5%)	***0.024***	98 (96.1%)	101 (89.4%)	*0.152*	86 (84.3%)	64 (63.4%)	***0.007***
Methadone		35 (34.3%)	46 (46.9%)	*0.311*	38 (37.3%)	70 (61.9%)	***0.011***	19 (18.6%)	14 (13.9%)	*0.491*
Morphine		NA	NA		2 (2.0%)	13 (11.5%)	***0.022***	94 (92.2%)	93 (92.1%)	*0.993*
*Psychiatric co-medication*										
Benzodiazepines		18 (17.6%)	20 (20.4%)	*0.875*	20 (19.6%)	38 (33.6%)	*0.058*	34 (33.3%)	41 (40.6%)	*0.421*
Antipsychotics		13 (13%)	14 (14%)	*0.925*	14 (14%)	32 (28%)	***0.028***	19 (19%)	27 (27%)	*0.336*
Methylphenidate or Dexmethylphenidate		NA	NA		0	9 (8.0%)	***0.022***	3 (2.9%)	11 (10.9%)	*0.069*
Antidepressants		NA	NA		22 (21.6%)	36 (31.9%)	*0.180*	27 (26.5%)	43 (42.6%)	*0.050*
*Additional substance use*										
Non-prescribed drug use		32 (31.4%)	27 (27.6%)	*0.831*	35 (34.3%)	46 (40.7%)	*0.479*	52 (51.0%)	69 (68.3%)	*0.050*
Problematic alcohol use		19 (18.6%)	22 (22.4%)	*0.831*	23 (22.5%)	30 (26.5%)	*0.575*	25 (24.5%)	30 (29.7%)	*0.524*
*Hepatitis C*										
HCV antibody positive		81 (79.4%)	78 (79.6%)	*0.999*	83 (81.4%)	80 (70.8%)	*0.156*	84 (82.4%)	68 (67.3%)	*0.050*
	HCV RNA positive	50/81 (61.7%)	54/78 (69.2%)	*0.621*	44/83 (53.0%)	48/80 (60.0%)	*0.479*	10/84 (11.9%)	19/68 (27.9%)	*0.050*
	HCV treated with SVR	8/81 (9.9%)	0	***0.024***	14/83 (16.9%)	3/80 (3.8%)	***0.022***	48/84 (57.1%)	28/68 (41.2%)	*0.122*
	HCV with spontaneous clearance	23/81 (28.4%)	24/78 (30.8%)	*0.999*	25/83 (30.1%)	29/80 (36.3%)	*0.496*	26/84 (31.0%)	21/68 (31.0%)	*0.993*
*HIV*										
Diagnosed		12 (11.8%)	12 (12.2%)	*0.999*	13 (12.8%)	10 (8.85%)	*0.479*	13 (12.8%)	6 (5.9%)	*0.211*
Treated if diagnosed		NA	NA		12 (92.3%)	10 (100%)	*0.479*	12 (92.3%)	6 (100%)	*0.593*

All values are median (interquartile range) or number (%). Abbreviations: HCV, hepatitis C virus; HIV, human immunodeficiency virus; SVR, sustained virological response. *p*-values in bold were significant after Benjamini Hochberg correction assuming a false discovery rate of 0.1.

^a)^ One long-term participant transitioned from female to male between 2012 and 2019.

The OAT prescription patterns were similar for long-term participants compared to non-long-term patients however, a higher proportion of long-term patients were still treated with heroin in 2019 (86/102 (84.3%) versus 64/101 (64.4%), *p* = 0.007)).

Non-prescribed drug use remained an important issue in this group; the number of patients who self-reported non-prescribed drug use increased from 32/102 (31%) in 2009, to 52/102 (51%) in 2019, but was lower than in other patients treated in 2019 (52/102 (51%) versus (69/101 (67%), *p* = 0.050). Problematic alcohol consumption also remained high, affecting 19/102 (18.6%) long-term patients in 2009 and 25/102 (24.5%) in 2019, similar to non-long-term participants.

Three HCV-antibody seroconversions occurred during the observation period: 81/102 (79.4%) patients were HCV-antibody positive in 2009, 83/102 (81.4%) in 2012 and 84/102 (82.4%) in 2019. However, the number of patients with detectable HCV RNA levels decreased significantly from 50/81 (61.7%) in 2009 to 10/84 (11.9%) in 2019, *p* = 0.004 (Supplementary Material, sTable 1). Among long-term participants in 2019, 26/84 (31%) had spontaneous clearance and 48/84 (57%) achieved a sustained virological response (SVR) after HCV treatment. Interestingly, although statistically not significant, a higher proportion of long-term participants were HCV antibody positive in 2019 compared to other patients (84/102 (82.4%) versus 68/101 (67.3%), *p* = 0.050) with a lower proportion of RNA positive patients (10/84 (11.9%) versus 19/68 (27.9%), *p* = 0.050). Comparing long-term participants to individuals only registered in 2019 (n = 72, Supplementary Material, sTable 2), confirmed these differences and also showed that a higher proportion of long-term participants were successfully treated for HCV compared to short-term patients (48/84 (57.1%), versus 15/44(31.1%) *p* = 0.041).

Among long-term participants, one new HIV infection was reported between 2009 and 2012 (12, 13, and 13 cases in 2009, 2012, and 2019, respectively). In 2019, 12/13 long-term participants were treated (one patient refused treatment based on personal beliefs). All patient on antiretroviral treatment had undetectable HIV RNA levels.

### 3.3 Medical comorbidities in HAT patients over time

A significant increase in diagnosed cardiovascular and musculoskeletal disease was observed over time (cardiovascular 26/200 (13.0%) in 2009 to 75/203 (37%) in 2019, *p* < 0.001; musculoskeletal 0 in 2009 to 32/203 (15.8%) in 2019, *p* < 0.001), (Table 3).

**Table 3:** Medical comorbidities over time, all participants.

Year of survey	2009	2012	2019	
	N = 200 (%)	N = 215 (%)	N = 203 (%)	*p*-value
*Comorbidity by organ system*				
Cardiovascular disease	26 (13.0%)	32 (14.9%)	75 (37.0%)	***< 0.001***
Lung disease	26 (13.0%)	18 (13.4%)	25 (12.3%)	*0.339*
Liver disease/cirrhosis	9 (4.5%)	16 (7.4%)	11 (5.4%)	*0.482*
Neurological disease	25 (12.5%)	26 (12.1%)	23 (11.3%)	*0.935*
Endocrinological disease	17 (8.5%)	18 (8.4%)	27 (13.3%)	*0.267*
Musculoskeletal diseases	0	2 (0.9%)	32 (15.8%)	***< 0.001***
*Number of comorbidities per patient*				
No comorbidities	121 (60.5%)	130 (60.5%)	89 (43.8%)	***< 0.001***
One comorbidity	58 (29.0%)	63 (29.3%)	59 (29.1%)	
Two comorbidities	18 (9.0%)	17 (7.9%)	37 (18.2%)	
Three comorbidities	3 (1.5%)	5 (2.3%)	13 (6.4%)	
Four comorbidities	0	0	4 (2.0%)	
Five comorbidities	0	0	1 (0.5%)	
Patients with multimorbidity (≥2 comorbidities)	21 (10.5%)	22 (10.2%)	55 (27.1%)	***< 0.001***

All values are number (%).

*p*-values in bold were significant after Benjamini Hochberg correction assuming a false discovery rate of 0.1

A significant shift in number of comorbidities per patient occurred over time with a decreasing number of patients with no medical comorbidities (121/200 (60.5%) in 2009, 130/215 (60.5%) in 2012 and 89/203 (43.8%) in 2019, *p* < 0.001).

The proportion of patients with multimorbidity (excluding psychiatric disorders), increased from 21/200 (10.5%) in 2009, to 55/203 (27.1%) in 2019 (*p* < 0.001).

In 2019, a significantly higher percentage of long-term participants suffered from multimorbidity compared to others (38/102 (37.3%) versus 17/101 (16.8%), *p* = 0.008) (Table 4 and Supplementary Material, sTable 3A). These differences were confirmed when comparing long-term participants to short-term patients only registered in 2019 (Supplementary Material, sTable 3B).

**Table 4:** Medical comorbidities over time, long term participants versus others.

Year of survey	2009			2012			2019		
Participant group	Long term	others		Long term	others		Long term	others	
	N = 102 (%)	N = 98 (%)	*p*-value	N = 102 (%)	113 (%)	*p*-value	N = 102 (%)	101 (%)	*p*-value
*Comorbidity by organ system*									
Cardiovascular disease	12 (11.8%)	14 (14.3)	*0.695*	11 (10.8%)	21 (18.6%)	*0.448*	42 (41.2%)	33 (32.7%)	*0.239*
Lung disease	8 (7.8%)	18 (18.4)	*0.189*	8 (7.8%)	10 (8.9%)	*0.790*	18 (17.6%)	7 (6.9%)	*0.068*
Liver disease/cirrhosis	5 (4.9%)	4 (4.1)	*0.780*	5 (4.9%)	11 (9.7%)	*0.448*	9 (8.8%)	2 (2.0%)	*0.068*
Neurological disease	14 (13.7%)	11 (11.2)	*0.695*	11 (10.8%)	15 (13.3%)	*0.658*	12 (11.8%)	11 (10.9%)	*0.844*
Endocrinological disease	7 (6.9%)	10 (10.2)	*0.695*	7 (6.9%)	11 (9.7%)	*0.597*	17 (16.7%)	10 (9.9%)	*0.208*
Musculoskeletal diseases	0	0		2 (2.0%)	0	*0.448*	21 (20.6%)	11 (10.9%)	*0.093*
*Number of comorbidities per patient*									
No comorbidities	66 (64.7%)	55 (56.1%)	*0.695*	67 (65.7%)	63 (55.8%)	*0.528*	38 (37.3%)	51 (50.5%)	*0.068*
One comorbidity	27 (26.5%)	31 (31.6%)		28 (27.5%)	35 (31.0%)		26 (25.5%)	33 (32.7%)	
Two comorbidities	8 (7.8%)	10 (10.2%)		5 (4.9%)	12 (10.6%)		25 (24.5%)	12 (11.9%)	
Three comorbidities	1 (1.0%)	2 (2.0%)		2 (2.0%)	3 (2.7%)		10 (9.8%)	3 (3.0%)	
Four comorbidities	0	0		0	0		2 (2.0%)	2 (2.0%)	
Five comorbidities	0	0		0	0		1 (1.0%)	0	
Patients with multimorbidity (≥2 comorbidities)	9 (8.8%)	12 (12.2%)	*0.695*	7 (6.9%)	15 (13.3%)	*0.448*	38 (37.3%)	17 (16.8%)	***0.008***

All values are number (%).

*p*-values in bold were significant after Benjamini Hochberg correction assuming a false discovery rate of 0.1

**Fig. 1:**
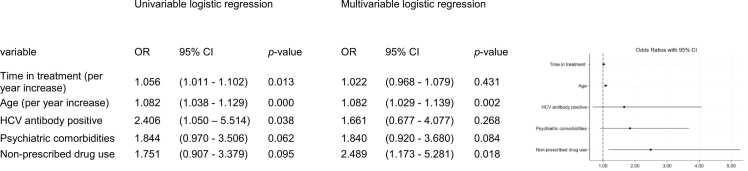
Factors associated with multimorbidity at final assessment.

In univariable analyses, age was a predictor of multimorbidity for the population registered in 2019 (OR 1.08, 95% CI 1.04–1.13, *p* < 0.001), (Fig. 1). Time in treatment was also significantly associated with multimorbidity (OR 1.06, 95% CI 1.01–1.10, *p* = 0.013), as was HCV seropositivity (OR 2.41, 95% CI 1.05–5.51, *p* = 0.038). Psychiatric comorbidity (OR 1.84, 95% CI 0.97–3.51, *p* = 0.062) and non-prescribed drug use (OR 1.75, 95% CI 0.91–3.38, *p* = 0.095) did not reach statistical significance in univariable analyses but were retained in the multivariable model on clinical grounds, as detailed in the Methods.

In the multivariable model, age remained a statistically significant independent predictor of multimorbidity (OR 1.08, 95% CI 1.03–1.14, *p* = 0.002) after adjustment for all other covariates. Non-prescribed drug use reached statistical significance in the multivariable model (OR 2.49, 95% CI 1.17–5.28, *p* = 0.018), suggesting that its effect had been masked by unadjusted confounding in the univariable analysis. (Fig. 1).

Assessment of collinearity among predictors did not reveal evidence of problematic multicollinearity (mean VIF = 1.30; range: 1.04–1.60; all tolerance values > 0.62; see Supplementary Material, sTable 4). The attenuation of the associations of time in treatment and HCV seropositivity following multivariable adjustment is therefore more consistent with confounding, most plausibly by age, than with multicollinearity.

## 4 Discussion

Based on repeated cross-sectional data collected over 10 years, this study characterizes long-term health patterns among people who use drugs (PWUD) treated at a specialized heroin-assisted opioid agonist therapy center in Switzerland, a population that has historically faced substantial barriers to healthcare (Heidari et al., 2023). While previous research has primarily focused on psychiatric comorbidities and infectious diseases such as HCV and HIV among individuals with opioid use disorder (Hasin and Kilcoyne, 2012, Kar et al., 2023, Wang et al., 2016), data on the prevalence and evolution of non-communicable diseases (NCDs) in this population remain limited.

Infectious disease outcomes in this study population reflect the success of sustained engagement in care and advances in treatment. Over the 10-year period, the prevalence of active HCV infection in long-term participants declined markedly, with HCV RNA positivity decreasing from 61.7% of antibody positive individuals in 2009 to 11.9% in 2019. This trend underscores the impact of direct-acting antivirals and long-term retention in harm-reduction based treatment (Graf et al., 2020, Grebely et al., 2015, Martinello et al., 2023). The higher prevalence of HCV antibody positivity in long-term participants in 2019 likely reflects cumulative lifetime exposure or differences in risk behaviors compared with more recently enrolled patients.

As the PWUD population ages, NCDs are emerging as major contributors to morbidity and mortality (Lewer et al., 2019). Although harm-reduction programs have substantially reduced infectious disease transmission and overdose deaths, chronic conditions such as cardiovascular, metabolic, and respiratory diseases have received comparatively less attention (Degenhardt et al., 2017, Degenhardt et al., 2023). Earlier studies have reported high rates of chronic illness and multimorbidity among PWUD engaged in OAT (Cullen et al., 2009), along with increasing mortality from cardiovascular disease, liver disease and cancer (Degenhardt et al., 2014, Larney et al., 2020).

Our findings are consistent with a recent Swiss study reporting a high prevalence and early onset of multimorbidity among individuals receiving various OAT modalities, with only a minority on heroin-assisted treatment (Lütolf et al., 2026). The present study extends this work by providing a detailed characterization of multimorbidity in a highly marginalized population specifically engaged in heroin-assisted treatment.

Age was strongly associated with multimorbidity in our study population consistent with previous reports (Lewer et al., 2019) and findings in the general population (Chowdhury et al., 2023, Pache et al., 2015). However, the prevalence of multimorbidity among individuals aged 50–59 years in the general population in Switzerland is estimated at 15.7% (CI 12.3.–19.1) (Rizza et al., 2012), whereas we observed a substantially higher prevalence of 37.3% among long-term participants in the same age group. This suggests a disproportionate burden of medical conditions in this study population. Similar associations have been described in other populations with opioid use disorder, where older age and multiple chronic conditions were linked to higher inpatient healthcare utilization (Han et al., 2022). These findings carry important clinical and public health implications. The OAT population in Switzerland and other high-income countries is aging, with a growing proportion of patients now aged 50 or older (Carew and Comiskey, 2018, Degenhardt et al., 2023, Lewer et al., 2019), and the prevalence of co-occurring chronic conditions is likely to grow. This highlights the need to proactively integrate evidence-based interventions for age-related chronic disease management into existing OAT frameworks (Fortin et al., 2007, Salisbury et al., 2018).

In contrast to Lütolf et al. (Lütolf et al., 2026), we did not observe a reduction in self-reported non-prescribed drug use over time among long-term participants. This discrepancy may reflect the greater social and clinical complexity of patients treated in heroin-assisted settings, or a lower threshold for reporting ongoing drug use in a familiar, long-standing care environment. Ongoing non-prescribed drug use was associated with multimorbidity in the multivariable model, which may reflect reduced engagement in preventive health behaviors and persistent barriers to care (Heidari et al., 2022, Heidari et al., 2023). These findings underscore the importance of addressing ongoing substance use as an integral component of chronic disease management in this population.

The substantial increase in diagnosed psychiatric comorbidities observed between 2009 and 2019 further underscores the complexity of this population. These trends likely reflect several non-mutually exclusive mechanisms: improved psychiatric screening within OAT settings; the demographic aging of the Swiss OAT population; and a potential shift toward a treatment-seeking population with greater psychiatric burden, as harm reduction policies have reduced opioid initiation in the general population (Khan et al., 2014, Lütolf et al., 2026). Irrespective of the underlying mechanism, patients with co-occurring substance use and mental disorders experience worse treatment outcomes, higher hospitalization rates and elevated suicide risk when both conditions are not addressed concurrently (Quello et al., 2005, Schulte and Hser, 2014, Torrens et al., 2011). Furthermore, multimorbidity patterns and healthcare-seeking behaviors in this population are shaped by psychiatric comorbidities and social determinants of health (Heidari et al., 2022).

Managing complex medical conditions in PWUD remains challenging. Many individuals avoid healthcare settings due to prior experiences of stigmatization, discrimination, or perceived low quality of care (Heidari et al., 2022, Heidari et al., 2023, Heidari et al., 2024, Wang et al., 2016, Friedmann et al., 2003, Myles et al., 2011). Negative provider attitudes and fragmented psychiatric and medical services further undermine continuity of care and treatment adherence (Messinger and Suzuki, 2022, Miller-Lloyd et al., 2020, Visconti et al., 2019). These barriers are particularly consequential given the growing psychiatric and medical complexity documented in this study.

Harm-reduction based approaches such as heroin-assisted or opioid agonist treatment with on-site medical and psychiatric services provide a structured framework to address the complex and intersecting healthcare needs of PWUD (Samet et al., 2001, Friedmann et al., 2003).

Overall, the marked increase in diagnosed medical and psychiatric comorbidities in long-term participants over the 10-year observation period highlights a transition from predominantly infectious to chronic, age-related disease burden. This shift, compounded by the demographic aging of the OAT population itself, emphasizes the growing need for comprehensive, integrated treatment strategies that address infectious diseases, non-communicable diseases and mental health conditions within coordinated care models tailored to aging PWUD.

## 5 Limitations

This study is a single-center descriptive analysis of patients enrolled in heroin-assisted treatment between 2009 and 2019. While the design enabled detailed observations over a 10-year period and a nuanced characterization of health patterns among long-term participants, several limitations should be acknowledged: First, the relatively small sample size precluded more complex inferential analyses. Second, the analysis was based on sequential cross-sectional assessments of patients actively registered at the treatment center at each survey time point and represents a survivor sample, introducing potential selection bias. Third, due to the cross-sectional nature of assessments, data on mortality and reasons for program discontinuation were not available, precluding outcome evaluations for patients who did not remain in long-term treatment.

The specialized care model at the study site, with integrated on-site medical services and an approximate 50% retention rate over 10 years, may have facilitated more coordinated care compared with less integrated settings. This may limit the generalizability of our findings to broader populations of PWUD or to other opioid agonist treatment modalities.

In addition, individuals requiring long-term HAT represent a distinct and highly marginalized subgroup of PWUD with substantial health vulnerabilities. Patterns of multimorbidity observed in this study population may therefore differ from populations with different demographic, clinical or social characteristics.

Important risk factors for several medical conditions including body mass index, blood pressure and smoking status were not systematically recorded in routine clinical documentation and therefore could not be incorporated in the analysis, limiting the ability to explore potential causal explanations.

Finally, although we observed an increasing prevalence of non-communicable diseases over time, it remains unclear whether this reflects a true increase in disease prevalence or improved diagnostic recognition among providers caring for patients with sustained treatment engagement. Similarly, the observed increases in psychiatric diagnoses may partly reflect changes in screening practices or documentation standards rather than true increases in prevalence. The descriptive nature of this study also precludes causal inference or evaluation of specific interventions aimed at preventing or reducing medical comorbidities.

## 6 Conclusion

As advances in antiviral therapy have led to declines in HCV and HIV prevalence, new challenges are emerging in the long-term care of people receiving OAT. As the population of PWUD ages, NCDs are becoming a dominant contributor to morbidity and healthcare demand. Integrated care models combining harm reduction with proactive management of chronic conditions may improve early detection, continuity of care, and ultimately optimize health outcomes in this medically and socially vulnerable population.

## Authors contributions

MCT study conceptualization, initial data collection, data cleaning and analysis manuscript review

DW and DS: Data collection, data analysis writing and reviewing of manuscript

PB, SL, and EH: data collection, validation, review of manuscript

## CRediT authorship contribution statement

**Sonja Loebnitz:** Writing – review & editing, Validation, Resources, Data curation. **Peer W. Brandt:** Writing – review & editing, Validation, Resources, Data curation. **Dario Willi:** Writing – original draft, Validation, Investigation, Formal analysis, Data curation. **David Strittmatter:** Writing – original draft, Validation, Investigation, Formal analysis, Data curation. **Maria Christine Thurnheer:** Writing – review & editing, Validation, Supervision, Methodology, Formal analysis, Conceptualization. **Eveline Hofmann:** Writing – review & editing, Validation, Resources, Investigation, Data curation.

## Consent for publication

N/A

## Ethics approval

Ethical considerations: The study proposal was reviewed by the Ethics Commission of the Canton of Berne, Switzerland, and was assessed as not requiring formal approval owing to its purely observational design and the absence of study-related interventions (BASEC Nr. Req-2018–00855). All patients treated in the KODA signed a general consent form for further use of the encoded data recorded during routine care.

## Funding

This study was funded by institutional money as part of institutiinal quality improvement efforts, no grants or other funding bodies were involved.

## Declaration of Competing Interest

The authors declare that they have no known competing financial interests or personal relationships that could have appeared to influence the work reported in this paper.

## Data Availability

The datasets generated and/or analyzed during the current study are not publicly available because of the sensitive nature of the encoded data, but are available from the corresponding author upon reasonable request.

## Glossary

ART: antiretroviral treatment (used for HIV treatment)
DAA: directly acting antiviral drugs (used for HCV treatment)
HAT: heroin-assisted treatment
HCV: hepatitis C virus
HIV: human immune deficiency virus
NCD: non-communicable disease
NPS: needle and syringe exchange programs
OAT: opioid agonist treatment
PWID: people who inject drugs
PWUD: people who use drugs
SVR: sustained virological response
