# Supplementary material for: Patterns of medical comorbidities among aging people receiving heroin-assisted treatment: A 10-year single-center repeated cross-sectional study
Source: Drug Alcohol Depend Rep. 2026 Apr 1;19:100435. doi: 10.1016/j.dadr.2026.100435 (PMC13091449; doi:10.1016/j.dadr.2026.100435)
Supplement: Supplementary material [file mmc1.docx]

| **sTable 1. Population characteristics over time: long-term participants** | | | | | |
| --- | --- | --- | --- | --- | --- |
| Year of survey | | 2009 | 2012 | 2019 |  |
|  | | N = 102 (%) | N = 102 (%) | N = 102 (%) | *p*-value |
| *Demographics* | |  |  |  |  |
| Age, years | | 40 (36-44) | 44 (40-48) | 52 (49-57) | **0.004** |
| Time in KODA, years | | 8 (5-11) | 11 (8-14) | 19 (16-22) | **0.004** |
| Women | | 30 (29.4%) | 29 (28.4%) ^a)^ | 29 (28.4%) | 1.000 |
|  | |  |  |  |  |
| *Psychiatric diagnosis* | |  |  |  |  |
| F3 Affective Disorders | | 25 (24.5%) | 27 (26.5%) | 35 (34.3%) | 0.438 |
| F6 Personality Disorder | | 22 (21.6%) | 22 (21.6%) | 32 (31.4%) | 0.319 |
| F2 Schizophrenia | | 3 (2.9%) | 4 (3.9%) | 15 (14.7%) | **0.004** |
| At least 1 psychiatric comorbidity (F3, F6 or F2) | | 42 (41.2%) | 47 (46.1%) | 52 (51%) | 0.581 |
|  | |  |  |  |  |
| *Opiate agonist treatment* | |  |  |  |  |
| Heroin | | 91 (89.2%) | 98 (96.1%) | 86 (84.3%) | **0.044** |
| Methadone | | 35 (34.3%) | 38 (37.3%) | 19 (18.6%) | **0.022** |
| Morphine | | NA | 2 (2.0%) | 94 (92.2%) | **0.004** |
|  | |  |  |  |  |
| *Psychiatric co-medication* | |  |  |  |  |
| Benzodiazepines | | 18 (17.6%) | 20 (19.6%) | 34 (33.3%) | **0.039** |
| Antipsychotics | | 13 (13%) | 14 (14%) | 19 (19%) | 0.622 |
| Methylphenidate or Dexmethylphenidate | | NA | 0 (0%) | 3 (2.9%) | 0.162 |
| Antidepressants | | NA | 22 (21.6%) | 27 (26.5%) | 0.606 |
|  | |  |  |  |  |
| *Additional substance use* | |  |  |  |  |
| Non-prescribed drug use | | 32 (31.4%) | 35 (34.3%) | 52 (51.0%) | **0.022** |
| Problematic alcohol use | | 19 (18.6%) | 23 (22.5%) | 25 (24.5%) | 0.758 |
|  | |  |  |  |  |
| *Hepatitis C* | |  |  |  |  |
| HCV antibody positive | | 81(79.4%) | 83 (81.4%) | 84 (82.4%) | 0.998 |
|  | HCV RNA positive | 50/81 (61.7%) | 44/83 (53.0%) | 10/84 (11.9%) | **0.004** |
|  | HCV treated with SVR | 8/81 (9.9%) | 14/83 (16.9%) | 48/84 (57.8%) | **0.004** |
|  | HCV with spontaneous clearance | 23/81 (28.4%) | 25/83 (30.1%) | 26/84 (30.9%) | 0.998 |
|  | |  |  |  |  |
| *HIV* | |  |  |  |  |
| Diagnosed | | 12 (11.8%) | 13 (12.8%) | 13 (12.8%) | 0.998 |
| Treated if diagnosed | | NA | 12 (92.3%) | 12 (92.3%) | 1.000 |
|  | |  |  |  |  |
| All values are median (interquartile range) or number (%).  Abbreviations: HCV, hepatitis C virus; HIV, human immunodeficiency virus; SVR, sustained virological response.   1. One individual transitioned from female to male gender   *p*-values in bold were significant after Benjamini Hochberg correction assuming a false discovery rate of 0.1 | | | | | |

**SUPPLEMENTARY MATERIAL**

| **sTable 2. Population characteristics at the final assessment: long-term versus short-term participants** | | | | |
| --- | --- | --- | --- | --- |
|  | | Long-term participants | Short-term participants |  |
|  | | N = 102 (%) | N = 72 (%) | *p*-value |
| *Demographics* | |  |  |  |
| Age, years | | 52 (49-57) | 42 (37-49) | **0.006** |
| Time in KODA, years | | 19.1 (16-22.2) | 4.6 (2-7) | **0.006** |
| Women | | 29 (28.4%) | 24 (33.3%) | 0.674 |
|  | |  |  |  |
| *Psychiatric diagnosis* | |  |  |  |
| F3 Affective Disorders | | 35 (34.1%) | 18 (25.0%) | 0.378 |
| F6 Personality Disorder | | 24 (23.5%) | 18 (25.0%) | 0.863 |
| F2 Schizophrenia | | 11 (10.8%) | 10 (13.9%) | 0.674 |
| At least 1 psychiatric comorbidity (F3, F6 or F2) | | 52 (51.0%) | 37 (51.4%) | 0.958 |
|  | |  |  |  |
| *Opiate agonist treatment* | |  |  |  |
| Heroin | | 86 (84.3%) | 42 (58.3%) | **0.006** |
| Methadone | | 19 (18.6%) | 8 (11.1%) | 0.378 |
| Morphine | | 94 (92.2%) | 67 (93.1%) | 0.863 |
|  | |  |  |  |
| *Psychiatric co-medication* | |  |  |  |
| Benzodiazepines | | 34 (33.3%) | 27 (37.5%) | 0.674 |
| Antipsychotics | | 19 (18.8%) | 17 (24.3%) | 0.666 |
| Methylphenidate or Dexmethylphenidate | | 3 (2.9%) | 6 (8.6%) | 0.279 |
| Antidepressants | | 27 (26.5%) | 24 (33.3%) | 0.553 |
|  | |  |  |  |
| *Additional substance use* | |  |  |  |
| Non-prescribed drug use | | 52 (51.0%) | 52 (72.2%) | **0.018** |
| Problematic alcohol use | | 25 (24.5%) | 26 (36.1%) | 0.270 |
|  | |  |  |  |
| *Hepatitis C* | |  |  |  |
| HCV antibody positive | | 84 (82.4%) | 44 (61.1%) | **0.009** |
|  | HCV RNA positive | 10/84 (11.9%) | 18/44 (40.9%) | **0.006** |
|  | HCV treated with SVR | 48/84 (57.1%) | 15/44 (34.1%) | **0.041** |
|  | HCV with spontaneous clearance | 26/84 (31%) | 11/44 (25%) | 0.674 |
|  | |  |  |  |
| *HIV* | |  |  |  |
| Diagnosed | | 13 (12.8%) | 5 (6.9%) | 0.396 |
| Treated if diagnosed | | 12/13 (92.3%) | 5/5 (100%) | 0.674 |
| All values are median (interquartile range) or number (%).  Abbreviations: HCV, hepatitis C virus; HIV, human immunodeficiency virus; SVR, sustained virological response.  *p*-values in bold were significant after Benjamini Hochberg correction assuming a false discovery rate of 0.1 | | | | |

| **sTable 3A. Medical comorbidities over time: long-term participants** | | | |  |
| --- | --- | --- | --- | --- |
| Year of survey | 2009 | 2012 | 2019 |  |
|  | N = 102 (%) | N = 102 (%) | N = 102 (%) | *p*-value |
| *Comorbidity by organ system* |  |  |  |  |
| Cardiovascular disease | 12 (11.8%) | 11 (10.8%) | 42 (41.2%) | **<0.001** |
| Lung disease | 8 (7.8%) | 8 (7.8%) | 18 (17.7%) | **0.037** |
| Liver disease/cirrhosis | 5 (4.9%) | 5 (4.9%) | 9 (8.8%) | 0.407 |
| Neurological disease | 14 (13.7%) | 11 (10.8%) | 12 (11.8%) | 0.806 |
| Endocrinological disease | 7 (6.9%) | 7 (6.9%) | 17 (16.7%) | 0.028 |
| Musculoskeletal diseases | 0 | 2 (1.9%) | 21 (20.6%) | **<0.001** |
|  |  |  |  |  |
| *Number of comorbidities per patient* |  |  |  |  |
| No comorbidities | 66 (64.7%) | 67 (65.6%) | 38 (37.3%) | **<0.0001** |
| One comorbidity | 27 (26.5%) | 28 (27.5%) | 26 (25.5%) |  |
| Two comorbidities | 8 (7.8%) | 5 (4.9%) | 25 (24.5%) |  |
| Three comorbidities | 1 (1%) | 2 (2%) | 10 (9.8%) |  |
| Four comorbidities | 0 | 0 | 2 (2%) |  |
| Five comorbidities | 0 | 0 | 1 (1%) |  |
|  |  |  |  |  |
| Patients with multimorbidity  (≥2 comorbidities) | 9 (8.8%) | 7 (6.86%) | 38 (37.3%) | **<0.0001** |
|  |  |  |  |  |
| All values are number (%).  *p*-values in bold were significant after Benjamini Hochberg correction assuming a false discovery rate of 0.1 | | | | |

| **sTable 3B. Medical comorbidities at the final assessment: long-term versus short term participants** | | | | |
| --- | --- | --- | --- | --- |
|  | Long-term participants | Short term participants |  |  |
|  | N=102 (%) | N=72 (%) |  | *p*-value |
| *Comorbidity by organ system* |  |  |  |  |
| Cardiovascular disease | 42 (41.2%) | 23 (31.9%) |  | 0.215 |
| Lung disease | 18 (17.7%) | 5 (6.9%) |  | **0.040** |
| Liver disease/cirrhosis | 9 (8.8%) | 0 |  | **0.010** |
| Neurological disease | 12 (11.8%) | 9 (12.5%) |  | 0.884 |
| Endocrinological disease | 17 (16.7%) | 5 (9.94%) |  | 0.057 |
| Musculoskeletal disease | 21 (20.6%) | 7 (9.7%) |  | 0.055 |
|  |  |  |  |  |
| *Number of comorbidities per patient* |  |  |  |  |
| No comorbidities | 38 (37.3%) | 39 (54.2%) |  | 0.057 |
| One comorbidity | 26 (25.5%) | 22 (30.6%) |  |  |
| Two comorbidities | 25 (24.5%) | 7 (9.7%) |  |  |
| Three comorbidities | 10 (9.8%) | 3 (4.1%) |  |  |
| Four comorbidities | 2 (2%) | 1 (1.4%) |  |  |
| Five comorbidities | 1 (1%) | 0 |  |  |
|  |  |  |  |  |
| Patients with multimorbidity  (≥2 comorbidities) | 38 (37.25%) | 11 (15.28%) |  | **0.002** |
|  |  |  |  |  |
| All values are number (%).  p-values in bold were significant after Benjamini Hochberg correction assuming a false discovery rate of 0.1 | | | | |

| **sTable 4. Multicollinearity Diagnostics** | | |
| --- | --- | --- |
|  |  |  |
| **Variable** | **VIF** | **1/VIF** |
| Time in treatment | 1.60 | 0.624447 |
| Age | 1.58 | 0.632990 |
| Non-prescribed drug use | 1.14 | 0.876351 |
| HCV seropositive | 1.13 | 0.886757 |
| Psychiatric comorbidity | 1.04 | 0.961096 |
| Mean VIF | 1.30 |  |
| Multicollinearity diagnostics were performed using an ordinary least squares regression model with identical predictors to the multivariable logistic regression model. VIF = Variance Inflation Factor. VIF > 10 and tolerance < 0.10 were predefined as indicative of serious multicollinearity. No predictor exceeded these thresholds. | | |
